# *Actinomyces meyeri* Empyema: A Case Report and Review of the Literature

**DOI:** 10.1155/2015/291838

**Published:** 2015-06-01

**Authors:** Hae Won Jung, Chong Rae Cho, Ji Yoon Ryoo, Hyun Kyo Lee, So Young Ha, Ji Hoon Choi, Yee Gyung Kwak

**Affiliations:** ^1^Department of Internal Medicine, Inje University Ilsan Paik Hospital, Goyang 411-706, Republic of Korea; ^2^Department of Laboratory Medicine, Inje University Ilsan Paik Hospital, Goyang 411-706, Republic of Korea; ^3^Department of Thoracic Surgery, Inje University Ilsan Paik Hospital, Goyang 411-706, Republic of Korea

## Abstract

*Actinomyces meyeri* is an uncommon cause of human actinomycosis. Here, we report a rare case of empyema caused by *A. meyeri*. A 49-year-old male presented with a history of 10 days of dyspnea and chest pain. A large amount of loculated pleural effusion was present on the right side and multiple lung nodules were documented on radiological studies. A chest tube was inserted and purulent pleural fluid was drained. *A. meyeri* was isolated in anaerobic cultures of the pleural fluid. The infection was alleviated in response to treatment with intravenous penicillin G (20 million IU daily) and oral amoxicillin (500 mg every 8 hours) for 4 months, demonstrating that short-term antibiotic treatment was effective.

## 1. Introduction

Actinomycosis is a chronic infection caused by bacteria belonging to the* Actinomyces* genus, with* Actinomyces israelii* most commonly implicated in human diseases, including cervicofacial disease. In contrast to other species of* Actinomyces*,* Actinomyces meyeri* often causes pulmonary infections and shows a tendency for hematogenous dissemination [1].* A. meyeri* infection of the respiratory system presents with nonspecific symptoms and has no characteristic findings of diagnostic value in radiological tests. Therefore, histological and microbiological tests are important for diagnosis. We herein report a case of empyema due to* A. meyeri* that was treated with short-term antibiotic therapy and review the current literature.

## 2. Case Report

**Figure 1:**
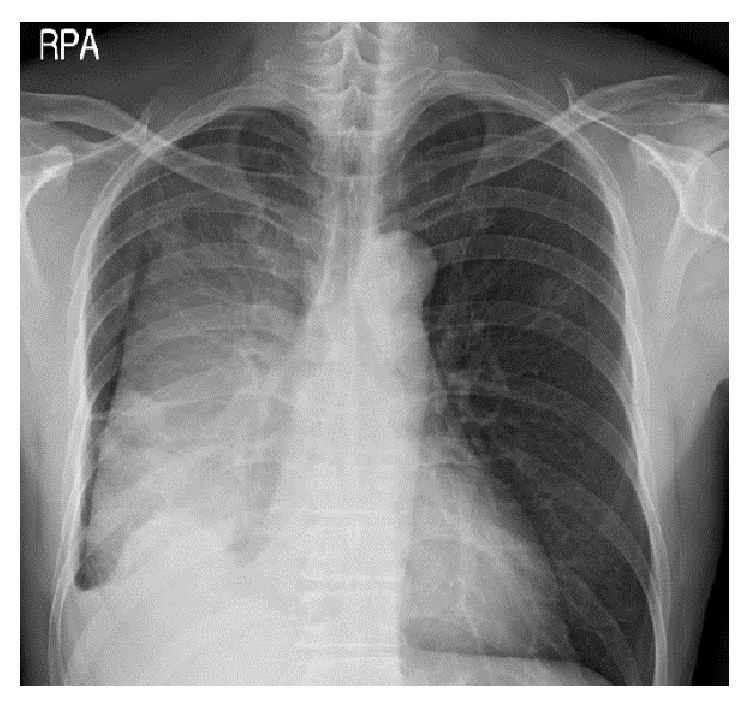
Large fissural loculated effusion in the right hemithorax.

**Figure 2:**
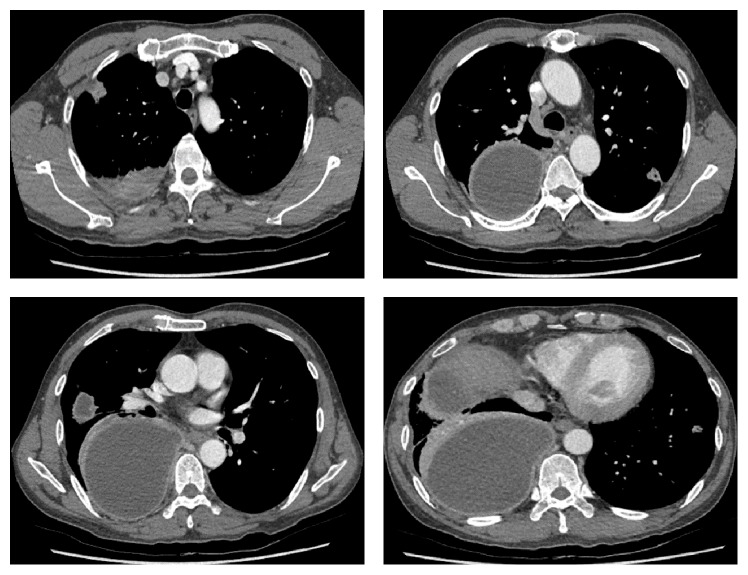
Multiple lung nodules and a large amount of multiloculated pleural effusion in the right hemithorax.

**Figure 3:**
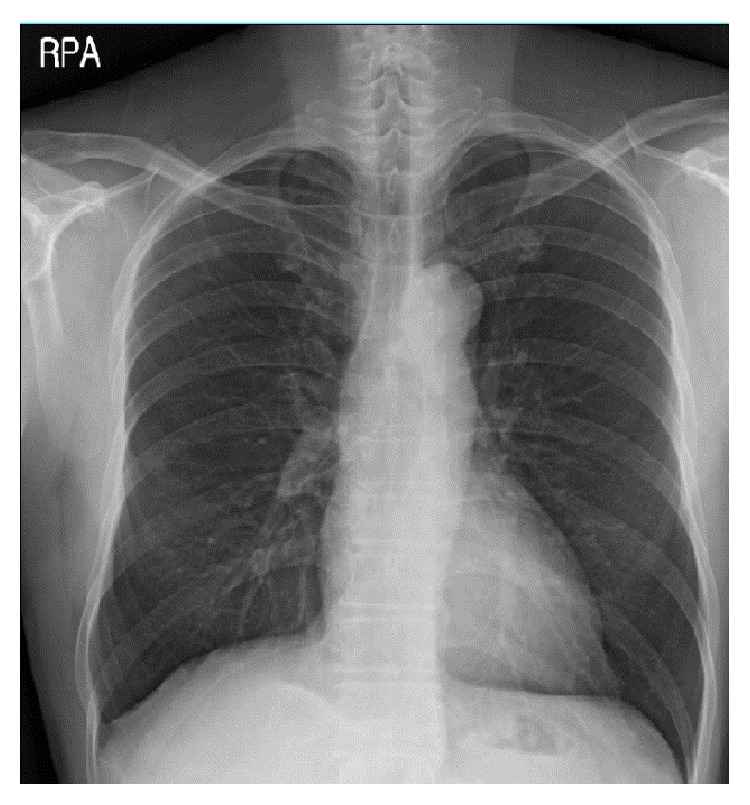
A chest radiograph showed the complete resolution of empyema without pleural sequelae 12 months after discontinuing treatment.

A 49-year-old male was hospitalized with a history of right chest pain occurring for 10 days and dyspnea. The patient had been in good health prior to this time and had no history of medical illness, except for smoking and alcohol abuse. The patient appeared acutely ill and a physical examination revealed that he was febrile (temperature 38.6°C). A chest examination revealed diminished breath sounds in the right side. Laboratory findings were significant for mild anemia (9.3 g/dL) and mild leukocytosis (12,260/mm^3^). Kidney and liver function tests were within normal limits. An arterial blood gas test without oxygen supply showed pH, partial pressure of oxygen, partial pressure of carbon dioxide, bicarbonate, and oxygen saturation readings of 7.472, 65.4 mmHg, 35.3 mmHg, 25.2 mmol/L, and 94%, respectively. A chest radiograph showed right side loculated pleural effusion (Figure 1). Computed tomography (CT) scans of the chest showed multiple lung nodules and right loculated pleural effusion (Figure 2). A chest tube was inserted and 1,380 mL of turbid yellow and pus-like pleural fluid was drained. A cell count of the pleural fluid revealed a white blood cell count of 56,000/mm^3^ with 89% polymorphonuclear leukocytes. The level of pleural lactate dehydrogenase was 20,530 IU/L and the levels of protein in the pleural fluid and serum were 3 g/dL and 5.9 g/dL, respectively. Gram stain of the pleural fluid specimen showed no organisms, and aerobic culture of the pleural fluid revealed no isolated pathogen. However, in anaerobic culture conditions, a 2 mm, elevated, gray colony was observed 48 hours after inoculation of the specimen onto phenylethyl alcohol agar. Gram stain of the colony growth showed a long, cribriform, non-spore-forming, Gram-positive* Bacillus* and this pathogen was ultimately identified as* A. meyeri* by the RAPID ID 32 A (bioMérieux, Marcy-l'Étoile, France) on the 10th day of hospitalization. The elapsed time from the collection of the pleural fluid specimen to incubation in an anaerobic environment was around 2 hours. From the day of admission, the patient was treated empirically with piperacillin/tazobactam (4.5 g IV every 8 hours) and the fever subsided after the third day of hospitalization. Antibiotics were changed to intravenous penicillin (20 million IU/day) after the pathogenic organism was identified as* A. meyeri*, and the course of penicillin treatment was 17 days. The symptoms were ameliorated and the lung nodules and pleural effusion were also improved on the follow-up chest radiograph and CT scan. The patient was discharged after hospitalization for 4 weeks and was prescribed oral amoxicillin (500 mg every 8 hours). After 8 weeks of oral amoxicillin therapy, a chest radiograph showed complete resolution of the empyema without pleural sequelae. The patient continued to receive amoxicillin therapy for approximately 5 more weeks to complete an almost 4-month course of antibiotic treatment and remained healthy during the 1-year follow-up period (Figure 3).

## 3. Discussion

Bacteria of the* Actinomyces* genus are anaerobic Gram-positive bacilli that typically reside in the mouth, gastrointestinal tract, and reproductive system. Disruption of the mucosal barrier is essential for the development of actinomycosis, which allows the organisms to invade. Cervicofacial and abdominal actinomycosis are associated with dental sepsis, appendicitis, diverticulitis, trauma, or surgery, while pelvic disease is related to intrauterine or intravaginal devices [1]. Pulmonary actinomycosis likely results from aspiration of oropharyngeal or gastrointestinal secretions into the respiratory tract and is initiated when saliva, or other material laden with* Actinomyces* species, is aspirated into a minor bronchus, causing atelectasis and pneumonitis [1].

Pulmonary actinomycosis frequently develops in middle- to old-aged males, especially those with a history of alcohol abuse and structural lung diseases [2]. Chest pain, productive cough, and dyspnea are the most common symptoms, and the most frequent radiologic feature is nonspecific consolidation. Although radiological tests may be helpful in diagnosing actinomycosis, differentiation from pneumonia or lung cancer is difficult. Direct isolation of the organism from a clinical specimen is necessary for a definitive diagnosis [3]. However, the failure rate of isolation is high (>50%) for various reasons, including previous antibiotic treatment, overgrowth of concomitant organisms, or inadequate methodology [3]. Most cases of actinomycosis reported to date had sulfur granules that were identified upon histological examination; however, sulfur granules may also be present in nocardiosis, coccidioidomycosis, and aspergillosis [4]. The difficulty of culturing and identifying* Actinomyces* has led to the use of new molecular genetic methods, such as 16s rRNA sequencing, which has facilitated more rapid and accurate identification of* Actinomyces* [3, 5, 6].

*A. meyeri* was first cultured from an empyema patient in 1911, but it is a very rare pathogen, with only 30 cases or so reported in the English-language literature to date [7, 8]. Unlike* A. israelii*,* A. meyeri* commonly infiltrates into the respiratory system, and it has the ability to spread to other organs such as the skin, long bones, liver, brain, and muscles. The propensity of* A. meyeri* to disseminate is difficult to explain because this organism does not differ from other* Actinomyces* species in its microbiological characteristics. It has been postulated that pulmonary infection is often the source of the hematogenous dissemination [9].

Actinomycosis in the respiratory system caused by* A. meyeri* may be effectively treated by antibiotics, similar to ordinary thoracic actinomycosis. Conventional therapy for actinomycosis is high-dose intravenous penicillin at a dosage of 18–24 million U/day for 2–6 weeks, followed by oral penicillin or amoxicillin for a period of 6–12 months [1]. However, the recommendations for the use of prolonged penicillin treatment date back to the early antibiotic era, and several studies have reported using shorter courses of antibiotics for actinomycosis [10]. A previous study described 19 patients exhibiting thoracic actinomycosis that were cured with a median duration of 6 weeks of antibiotic treatment (range, 1 week to 6 months) [11]. Another study including 16 patients reported an alleviation of symptoms in response to treatment consisting of a median duration of 2 weeks of intravenous penicillin and three months of oral penicillin [12]. These reports suggest that thoracic actinomycosis can be treated with relatively brief courses of antibiotic treatment.

A review of the English-language literature revealed five case reports of* A. meyeri* empyema [7, 8, 13–15] (Table 1). Four of five patients underwent a surgical procedure, and the duration of antibiotic therapy ranged from 6 to 12 months. In comparison to previous reports, the current case was diagnosed early and was effectively drained with a chest tube only. Additionally, there was no evidence of dissemination and symptoms and radiological findings were rapidly improved. On the basis of a clinical response, antibiotic treatment was maintained for a total of 4 months, and no evidence of recurrence was observed during the 1-year follow-up period. The duration of antibiotic treatment of actinomycosis depends on the initial burden of disease, the performance of resectional surgery, and the patient's response to treatment [3]. The traditional recommendation of 6–12 months of antibiotic therapy may not be necessary for all patients.

In conclusion, empyema due to* A. meyeri* is uncommon, and anaerobic culture of pleural fluid plays a major role in the early diagnosis of actinomycosis involving pleura. Although the loculated pleural effusion was large, early diagnosis and successful drainage could shorten the duration of treatment. Short-term antibiotic therapeutic treatment of actinomycosis can be attempted when the clinical and radiological responses are rapid and there is no evidence of dissemination.

## Figures and Tables

**Table 1:** Summary of six cases of empyema due to *A. meyeri*.

Case	Author (year) [reference]	Sex/age	Dissemination	Treatment	Antibiotic duration (months)
1	Rose et al. (1982) [13]	M/62	Hip	Chest tube drainage	13
2	Lentino et al. (1985) [14]	M/16	Bone marrow	Thoracotomy, resection of infected rib	6
3	Vallet et al. (2004) [15]	F/64	None	Thoracotomy and decortication	6.5
4	Fazili et al. (2012) [7]	M/45	None	Thoracotomy and decortication	12
5	Attaway and Flynn (2013) [8]	M/61	None	Thoracotomy and decortication	7.5
6	Present	M/49	None	Chest tube drainage	4
